# *N*′-(Diphenyl­methyl­ene)-2-hydroxy­benzo­hydrazide

**DOI:** 10.1107/S1600536809006916

**Published:** 2009-03-06

**Authors:** Yanfei Li, Chunhua Chen, Rongdong Yang, Rengao Zhao

**Affiliations:** a Department of Materials and Chemical Engineering, Taishan University, 271021 Taian, Shandong, People’s Republic of China; b No. 1 Middle School of Qufu, 273100, Shandong, People’s Republic of China

## Abstract

In the title compound, C_20_H_16_N_2_O_2_, intra­molecular N—H⋯O and inter­molecular O—H⋯O hydrogen bonds are found. The inter­molecular hydrogen bonds link the mol­ecules into an infinite chain along the *c* axis. The dihedral angles between the aromatic rings are 16.9 (3), 80.8 (3) and 64.6 (3)°

## Related literature

For the multiple-coordination environment of 2-hydroxybenzohydrazide and its derivatives, see: Chang (2008 ▶); Huo *et al.* (2004 ▶).
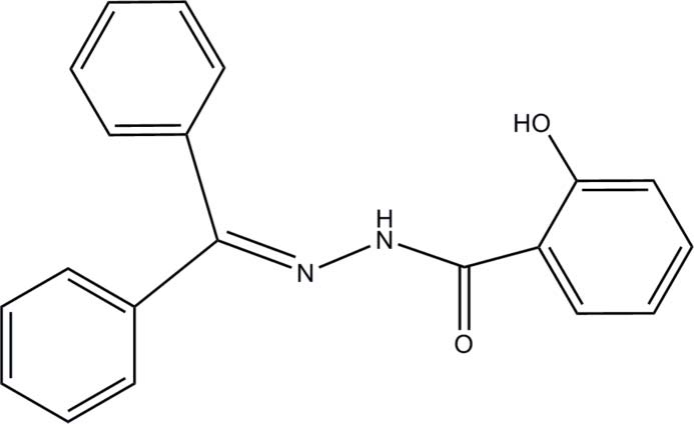


## Experimental

### 

#### Crystal data

C_20_H_16_N_2_O_2_
*M* *_r_* = 316.35
Tetragonal, 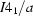

*a* = 16.5157 (9) Å
*c* = 24.401 (3) Å
*V* = 6655.8 (10) Å^3^
*Z* = 16
Mo *K*α radiation
μ = 0.08 mm^−1^
*T* = 273 K
0.31 × 0.25 × 0.19 mm

#### Data collection

Bruker SMART CCD area-detector diffractometer
Absorption correction: multi-scan (*SADABS*; Sheldrick, 1996 ▶) *T*_min_ = 0.972, *T*_max_ = 0.987
17393 measured reflections
2929 independent reflections
1610 reflections with *I* > 2σ(*I*)
*R*_int_ = 0.096

#### Refinement

*R*[*F* ^2^ > 2σ(*F* ^2^)] = 0.072
*wR*(*F* ^2^) = 0.255
*S* = 1.01
2929 reflections
217 parameters
7 restraints
H-atom parameters constrained
Δρ_max_ = 0.53 e Å^−3^
Δρ_min_ = −0.47 e Å^−3^

### 

Data collection: *SMART* (Siemens, 1996 ▶); cell refinement: *SAINT* (Siemens, 1996 ▶); data reduction: *SAINT*; program(s) used to solve structure: *SHELXS97* (Sheldrick, 2008 ▶); program(s) used to refine structure: *SHELXL97* (Sheldrick, 2008 ▶); molecular graphics: *SHELXTL* (Sheldrick, 2008 ▶); software used to prepare material for publication: *SHELXTL*.

## Supplementary Material

Crystal structure: contains datablocks I, global. DOI: 10.1107/S1600536809006916/rk2128sup1.cif

Structure factors: contains datablocks I. DOI: 10.1107/S1600536809006916/rk2128Isup2.hkl

Additional supplementary materials:  crystallographic information; 3D view; checkCIF report

## Figures and Tables

**Table 1:** Hydrogen-bond geometry (Å, °)

*D*—H⋯*A*	*D*—H	H⋯*A*	*D*⋯*A*	*D*—H⋯*A*
N2—H2*B*⋯O2	0.86	1.95	2.635 (3)	136
O2—H2*A*⋯O1^i^	0.82	1.87	2.688 (3)	172

Symmetry code: (i) 
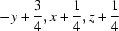
.

**Fig. 1.**
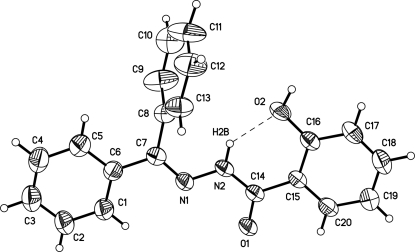
The molecular structure of the title compound with the atom numbering scheme. The displacement ellipsoids are shown at 30% probability level. H atoms are presented as a small spheres of arbitrary radius. Intramolecular H–bond shown by dashed line.

**Fig. 2.**
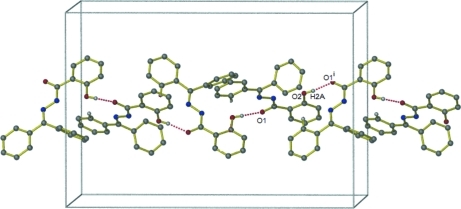
The one–dimensional chains are formed by the intermolecular H–bonds O2—H2···O1i. Symmetry code: (i) 3/4-y, 1/4+x, 1/4+z.

## supplementary crystallographic information

### Comment

The chemistry of 2-hydroxybenzohydrazide and its derivatives are studied because of their multiply coordination environment (Chang, 2008; Huo *et al.*, 2004). They represent a class of highly useful compounds in which the presence of O and N atom renders various hydrogen bonding motifs leading to the formation of versatile architecture in the crystal lattice. As the continue of the aspect, we study the reaction of benzophenone and 2–hydroxybenzohydrazide in the state of refluxing in ethanol.

Herein we report the molecular (Fig. 1) and crystal structures of the title compound, C_20_H_16_N_2_O_2_, which was characterized by elemental analyses too. In the structure an intramolecular N2—H2B···O2 (Fig. 1) and an intermolecular O2—H2A···O1^i^ (Fig. 2) H–bonds were found. Symmetry code: (i) 3/4-*y*, 1/4+*x*, 1/4+*z*).

### Experimental

Benzophenone and 2–hydroxybenzohydrazide were added to the solvent of ethanol and the mixture was stirred for 4 h at 323 K. After cooling down to the room temperature, the solution was filtered. The solvent was removed from the filtrate under vacuum and the solid residue was recrystallized from ether; colourless crystals suitable for X–ray diffraction study were obtained. Yield, 79%. m.p. 463 K. Analysis, calculated for C_20_H_16_N_2_O_2_: C 79.19, H 5.65, N 4.62; found: C 79.36, H 5.43, N 4.35%. The elemental analyses were performed with a Perkine Elemer PE2400II instrument.

### Refinement

The all H atoms were placed in idealized positions and constrained to ride on their parent atoms, with distances: N—H = 0.86 Å and O—H = 0.82 Å, C—H = 0.93 Å. The *U*_iso_(H) values were set at 1.2*U*_eq_(parent C, N) and 1.5*U*_eq_(O).

### Crystal data

**Table:**

C_20_H_16_N_2_O_2_	*D*_x_ = 1.263 Mg m^−^^3^
*M**_r_* = 316.35	Mo *K*α radiation, λ = 0.71073 Å
Tetragonal, *I*4_1_/*a*	Cell parameters from 3007 reflections
Hall symbol: -I 4ad	θ = 2.4–20.0°
*a* = 16.5157 (9) Å	µ = 0.08 mm^−^^1^
*c* = 24.401 (3) Å	*T* = 273 K
*V* = 6655.8 (10) Å^3^	Block, colourless
*Z* = 16	0.31 × 0.25 × 0.19 mm
*F*(000) = 2672	

### Data collection

**Table:**

Bruker SMART CCD area-detector diffractometer	2929 independent reflections
Radiation source: Fine–focus sealed tube	1610 reflections with *I* > 2σ(*I*)
Graphite	*R*_int_ = 0.096
φ and ω scans	θ_max_ = 25.0°, θ_min_ = 1.5°
Absorption correction: multi-scan (*SADABS*; Sheldrick, 1996)	*h* = −19→17
*T*_min_ = 0.972, *T*_max_ = 0.987	*k* = −19→19
17393 measured reflections	*l* = −24→28

### Refinement

**Table:**

Refinement on *F*^2^	Primary atom site location: Direct
Least-squares matrix: Full	Secondary atom site location: Difmap
*R*[*F*^2^ > 2σ(*F*^2^)] = 0.072	Hydrogen site location: Geom
*wR*(*F*^2^) = 0.255	H-atom parameters constrained
*S* = 1.01	*w* = 1/[σ^2^(*F*_o_^2^) + (0.158*P*)^2^ + 0.5238*P*] where *P* = (*F*_o_^2^ + 2*F*_c_^2^)/3
2929 reflections	(Δ/σ)_max_ = 0.001
217 parameters	Δρ_max_ = 0.53 e Å^−^^3^
7 restraints	Δρ_min_ = −0.47 e Å^−^^3^

### Special details

**Table:**

Geometry. All s.u.'s (except the s.u. in the dihedral angle between two l.s. planes) are estimated using the full covariance matrix. The cell s.u.'s are taken into account individually in the estimation of s.u.'s in distances, angles and torsion angles; correlations between s.u.'s in cell parameters are only used when they are defined by crystal symmetry. An approximate (isotropic) treatment of cell s.u.'s is used for estimating s.u.'s involving l.s. planes.
Refinement. Refinement of *F*^2^ against ALL reflections. The weighted *R*–factor *wR* and goodness of fit *S* are based on *F*^2^, conventional *R*–factors *R* are based on *F*, with *F* set to zero for negative *F*^2^. The threshold expression of *F*^2^ > σ(*F*^2^) is used only for calculating *R*–factors(gt) *etc*. and is not relevant to the choice of reflections for refinement. *R*–factors based on *F*^2^ are statistically about twice as large as those based on *F*, and *R*–factors based on ALL data will be even larger.

### Fractional atomic coordinates and isotropic or equivalent isotropic displacement parameters (Å^2^)

**Table:**

	*x*	*y*	*z*	*U*_iso_*/*U*_eq_	
N1	0.19770 (16)	0.48883 (16)	0.91131 (10)	0.0673 (8)	
N2	0.20636 (16)	0.53442 (16)	0.95782 (10)	0.0660 (8)	
H2B	0.1828	0.5197	0.9876	0.079*	
O1	0.28234 (15)	0.62777 (14)	0.91455 (9)	0.0834 (8)	
O2	0.18133 (14)	0.55740 (15)	1.06333 (8)	0.0770 (7)	
H2A	0.1681	0.5493	1.0953	0.115*	
C1	0.1847 (2)	0.3933 (2)	0.81748 (16)	0.0892 (12)	
H1	0.2122	0.4422	0.8148	0.107*	
C2	0.1798 (3)	0.3438 (3)	0.77280 (18)	0.1029 (13)	
H2	0.2036	0.3596	0.7399	0.123*	
C3	0.1404 (3)	0.2713 (3)	0.7760 (2)	0.1021 (14)	
H3	0.1382	0.2373	0.7456	0.123*	
C4	0.1046 (3)	0.2494 (3)	0.8232 (2)	0.1024 (14)	
H4	0.0765	0.2007	0.8251	0.123*	
C5	0.1094 (2)	0.2984 (2)	0.86910 (18)	0.0894 (12)	
H5	0.0855	0.2819	0.9018	0.107*	
C6	0.1494 (2)	0.3717 (2)	0.86662 (14)	0.0696 (9)	
C7	0.15649 (19)	0.4228 (2)	0.91578 (13)	0.0655 (9)	
C8	0.1194 (2)	0.3957 (2)	0.96790 (14)	0.0708 (9)	
C9	0.0389 (3)	0.4050 (3)	0.9779 (2)	0.1276 (19)	
H9	0.0055	0.4280	0.9515	0.153*	
C10	0.0067 (4)	0.3801 (3)	1.0278 (3)	0.1355 (17)	
H10	−0.0488	0.3835	1.0341	0.163*	
C11	0.0576 (4)	0.3502 (4)	1.0679 (2)	0.150 (2)	
H11	0.0368	0.3385	1.1024	0.180*	
C12	0.1362 (4)	0.3380 (3)	1.0581 (2)	0.1236 (18)	
H12	0.1693	0.3145	1.0845	0.148*	
C13	0.1676 (3)	0.3610 (3)	1.00770 (17)	0.0944 (13)	
H13	0.2223	0.3528	1.0006	0.113*	
C14	0.2516 (2)	0.6023 (2)	0.95709 (12)	0.0625 (8)	
C15	0.26762 (18)	0.64251 (19)	1.01038 (12)	0.0585 (8)	
C16	0.2366 (2)	0.6184 (2)	1.06129 (12)	0.0648 (9)	
C17	0.2630 (3)	0.6562 (3)	1.10856 (14)	0.0864 (11)	
H17	0.2436	0.6389	1.1423	0.104*	
C18	0.3167 (3)	0.7181 (3)	1.10648 (16)	0.0967 (13)	
H18	0.3334	0.7430	1.1388	0.116*	
C19	0.3466 (2)	0.7445 (3)	1.05717 (16)	0.0904 (12)	
H19	0.3830	0.7874	1.0557	0.108*	
C20	0.3218 (2)	0.7066 (2)	1.01018 (14)	0.0752 (10)	
H20	0.3422	0.7246	0.9768	0.090*	

### Atomic displacement parameters (Å^2^)

**Table:**

	*U*^11^	*U*^22^	*U*^33^	*U*^12^	*U*^13^	*U*^23^
N1	0.0836 (19)	0.0705 (17)	0.0477 (15)	−0.0095 (15)	0.0066 (13)	−0.0014 (12)
N2	0.0788 (18)	0.0773 (18)	0.0418 (14)	−0.0052 (14)	0.0106 (12)	0.0011 (12)
O1	0.1105 (19)	0.0922 (17)	0.0476 (13)	−0.0258 (14)	0.0190 (12)	−0.0067 (12)
O2	0.0928 (17)	0.0963 (17)	0.0417 (12)	−0.0020 (14)	0.0095 (11)	0.0066 (11)
C1	0.107 (3)	0.090 (3)	0.070 (3)	−0.021 (2)	0.003 (2)	−0.007 (2)
C2	0.128 (4)	0.108 (3)	0.073 (3)	−0.017 (3)	0.003 (2)	−0.024 (2)
C3	0.112 (3)	0.099 (3)	0.096 (3)	−0.005 (3)	−0.015 (3)	−0.027 (3)
C4	0.116 (3)	0.080 (3)	0.111 (4)	−0.016 (2)	−0.005 (3)	−0.015 (3)
C5	0.097 (3)	0.080 (3)	0.092 (3)	−0.011 (2)	0.003 (2)	−0.001 (2)
C6	0.070 (2)	0.069 (2)	0.070 (2)	−0.0040 (17)	−0.0008 (17)	−0.0007 (17)
C7	0.066 (2)	0.070 (2)	0.060 (2)	0.0028 (17)	0.0038 (15)	0.0030 (16)
C8	0.070 (2)	0.067 (2)	0.076 (2)	0.0049 (17)	0.0110 (17)	0.0075 (17)
C9	0.086 (3)	0.126 (4)	0.171 (4)	0.035 (3)	0.035 (3)	0.065 (3)
C10	0.1334 (19)	0.1375 (19)	0.1356 (19)	0.0024 (10)	0.0091 (10)	0.0072 (10)
C11	0.153 (5)	0.165 (5)	0.132 (5)	0.029 (4)	0.076 (4)	0.063 (4)
C12	0.145 (5)	0.131 (4)	0.095 (4)	0.007 (3)	0.021 (3)	0.047 (3)
C13	0.085 (3)	0.114 (3)	0.084 (3)	0.004 (2)	0.010 (2)	0.030 (2)
C14	0.0672 (19)	0.072 (2)	0.0485 (18)	0.0027 (17)	0.0073 (15)	−0.0005 (15)
C15	0.0619 (18)	0.0671 (19)	0.0464 (17)	0.0090 (16)	0.0051 (13)	−0.0028 (14)
C16	0.069 (2)	0.078 (2)	0.0472 (18)	0.0135 (18)	0.0011 (15)	0.0014 (15)
C17	0.110 (3)	0.104 (3)	0.0447 (19)	0.004 (3)	0.0011 (19)	−0.0018 (18)
C18	0.115 (3)	0.112 (3)	0.063 (3)	0.003 (3)	−0.008 (2)	−0.021 (2)
C19	0.098 (3)	0.097 (3)	0.076 (3)	−0.011 (2)	−0.001 (2)	−0.017 (2)
C20	0.080 (2)	0.088 (2)	0.058 (2)	0.005 (2)	0.0109 (17)	−0.0070 (18)

### Geometric parameters (Å, °)

**Table:**

N1—C7	1.289 (4)	C8—C13	1.380 (5)
N1—N2	1.370 (3)	C9—C10	1.390 (7)
N2—C14	1.347 (4)	C9—H9	0.9300
N2—H2B	0.8600	C10—C11	1.382 (8)
O1—C14	1.230 (4)	C10—H10	0.9300
O2—C16	1.361 (4)	C11—C12	1.336 (7)
O2—H2A	0.8200	C11—H11	0.9300
C1—C2	1.365 (5)	C12—C13	1.387 (6)
C1—C6	1.380 (5)	C12—H12	0.9300
C1—H1	0.9300	C13—H13	0.9300
C2—C3	1.365 (6)	C14—C15	1.484 (4)
C2—H2	0.9300	C15—C20	1.387 (5)
C3—C4	1.345 (6)	C15—C16	1.401 (4)
C3—H3	0.9300	C16—C17	1.382 (5)
C4—C5	1.384 (6)	C17—C18	1.354 (6)
C4—H4	0.9300	C17—H17	0.9300
C5—C6	1.381 (5)	C18—C19	1.372 (5)
C5—H5	0.9300	C18—H18	0.9300
C6—C7	1.472 (4)	C19—C20	1.369 (5)
C7—C8	1.481 (4)	C19—H19	0.9300
C8—C9	1.360 (5)	C20—H20	0.9300
			
C7—N1—N2	116.7 (3)	C11—C10—H10	120.3
C14—N2—N1	120.3 (2)	C9—C10—H10	120.3
C14—N2—H2B	119.9	C12—C11—C10	121.2 (5)
N1—N2—H2B	119.9	C12—C11—H11	119.4
C16—O2—H2A	109.5	C10—C11—H11	119.4
C2—C1—C6	121.0 (4)	C11—C12—C13	118.8 (5)
C2—C1—H1	119.5	C11—C12—H12	120.6
C6—C1—H1	119.5	C13—C12—H12	120.6
C1—C2—C3	120.5 (4)	C8—C13—C12	121.4 (4)
C1—C2—H2	119.7	C8—C13—H13	119.3
C3—C2—H2	119.7	C12—C13—H13	119.3
C4—C3—C2	119.6 (4)	O1—C14—N2	121.7 (3)
C4—C3—H3	120.2	O1—C14—C15	120.9 (3)
C2—C3—H3	120.2	N2—C14—C15	117.4 (3)
C3—C4—C5	120.7 (4)	C20—C15—C16	117.1 (3)
C3—C4—H4	119.6	C20—C15—C14	117.0 (3)
C5—C4—H4	119.6	C16—C15—C14	125.8 (3)
C6—C5—C4	120.3 (4)	O2—C16—C17	121.1 (3)
C6—C5—H5	119.9	O2—C16—C15	119.2 (3)
C4—C5—H5	119.9	C17—C16—C15	119.7 (3)
C1—C6—C5	117.8 (3)	C18—C17—C16	121.1 (4)
C1—C6—C7	121.8 (3)	C18—C17—H17	119.5
C5—C6—C7	120.4 (3)	C16—C17—H17	119.5
N1—C7—C6	117.3 (3)	C17—C18—C19	120.6 (4)
N1—C7—C8	123.2 (3)	C17—C18—H18	119.7
C6—C7—C8	119.5 (3)	C19—C18—H18	119.7
C9—C8—C13	118.9 (4)	C20—C19—C18	118.8 (4)
C9—C8—C7	121.6 (4)	C20—C19—H19	120.6
C13—C8—C7	119.4 (3)	C18—C19—H19	120.6
C8—C9—C10	119.9 (5)	C19—C20—C15	122.6 (3)
C8—C9—H9	120.0	C19—C20—H20	118.7
C10—C9—H9	120.0	C15—C20—H20	118.7
C11—C10—C9	119.5 (5)		

### Hydrogen-bond geometry (Å, °)

**Table:**

*D*—H···*A*	*D*—H	H···*A*	*D*···*A*	*D*—H···*A*
N2—H2B···O2	0.86	1.95	2.635 (3)	136
O2—H2A···O1^i^	0.82	1.87	2.688 (3)	172

Symmetry codes: (i) −*y*+3/4, *x*+1/4, *z*+1/4.
